# Obesogens: An Environmental Link to Obesity

**DOI:** 10.1289/ehp.120-a62

**Published:** 2012-02-01

**Authors:** Wendee Holtcamp

**Affiliations:** Houston-based freelancer Wendee Holtcamp has written for *Nature*, *Scientific American*, *National Wildlife*, and other magazines.


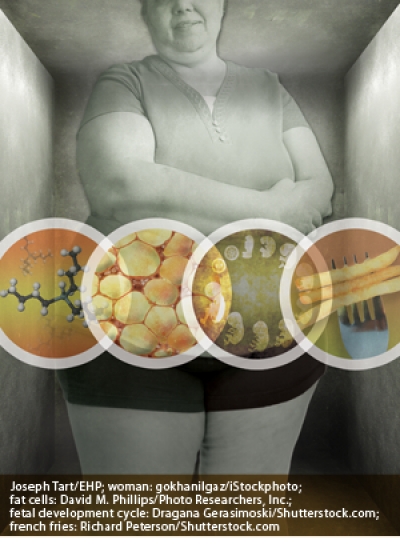
Obesity has risen steadily in the United States over the past 150 years,^1^ with a marked uptick in recent decades.^2^ In the United States today more than 35% of adults^3^ and nearly 17% of children aged 2–19 years are obese.^4^ Obesity plagues people not just in the United States but worldwide, including, increasingly, developing countries.^5^ Even animals—pets, laboratory animals, and urban rats—have experienced increases in average body weight over the past several decades,^6^ trends not necessarily explained by diet and exercise. In the words of Robert H. Lustig, a professor of clinical pediatrics at the University of California, San Francisco, “[E]ven those at the lower end of the BMI [body mass index] curve are gaining weight. Whatever is happening is happening to everyone, suggesting an environmental trigger.”^7^

Many in the medical and exercise physiology communities remain wedded to poor diet and lack of exercise as the sole causes of obesity. However, researchers are gathering convincing evidence of chemical “obesogens”—dietary, pharmaceutical, and industrial compounds that may alter metabolic processes and predispose some people to gain weight.^8^^,^^9^

Obesity is rising steadily around the world. Convincing evidence suggests that diet and activity level are not the only factors in this trend—chemical “obesogens” may alter human metabolism and predispose some people to gain weight. Fetal and early-life exposures to certain obesogens may alter some individuals’ metabolism and fat-cell makeup for life. Other obesogenic effects are linked to adulthood exposures.

Joseph Tart/EHP; woman: gokhanilgaz/iStockphoto; fat cells: David M. Phillips/Photo Researchers, Inc.; fetal development cycle: Dragana Gerasimoski/Shutterstock.com; french fries: Richard Peterson/Shutterstock.com

The idea that chemicals in the environment could be contributing to the obesity epidemic is often credited to an article by Paula Baillie-Hamilton, published in the *Journal of Alternative and Complementary Medicine* in 2002.^10^ Her article presented evidence from earlier toxicologic studies published as far back as the 1970s in which low-dose chemical exposures were associated with weight gain in experimental animals. At the time, however, the original researchers did not focus on the implications of the observed weight gains.

The role of environmental chemicals in obesity has garnered increased attention in academic and policy spheres, and was recently acknowledged by the Presidential Task Force on Childhood Obesity^11^ and the National Institutes of Health (NIH) Strategic Plan for Obesity Research.^12^ “Over the past ten years, and especially the past five years, there’s been a flurry of new data,” says Kristina Thayer, director of the Office of Health Assessment and Translation at the National Toxicology Program (NTP). “There are many studies in both humans and animals. The NTP found real biological plausibility.” In 2011 the NIH launched a 3-year effort to fund research exploring the role of environmental chemical exposures in obesity, type 2 diabetes mellitus, and metabolic syndrome.^13^

**Figure:**
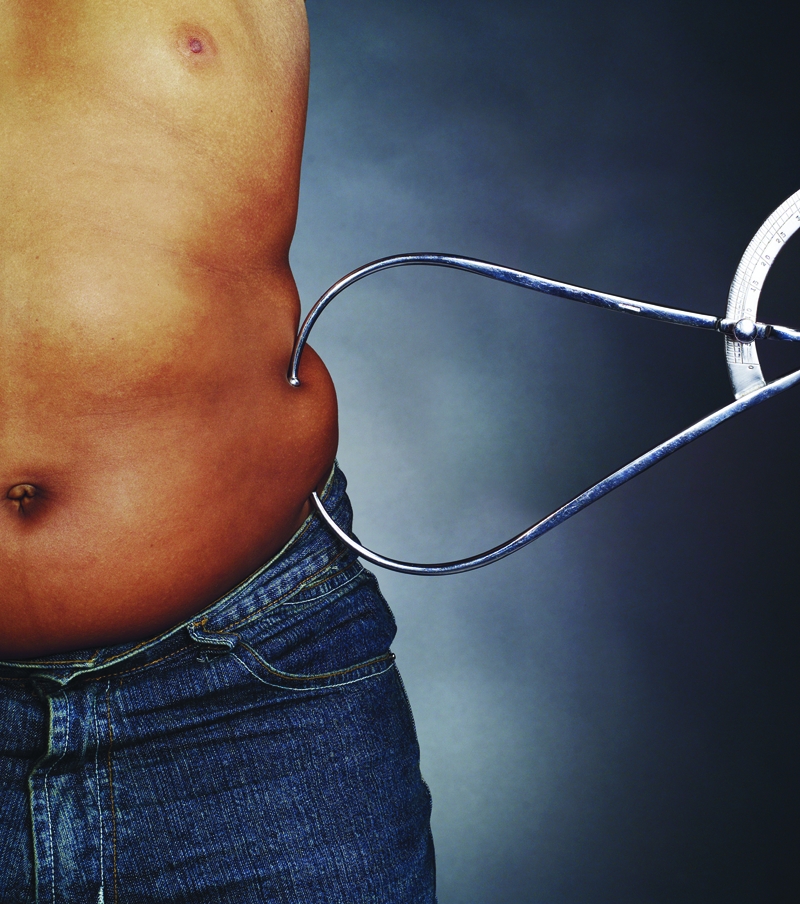
What Are Obesity and Overweight? For adults obesity is defined as having a BMI of 30 or more, whereas overweight is defined as having a BMI of 25 or more.^49^ Defining obesity is a bit more complicated for children; it depends on the age and sex of the child. Children are considered obese if they are at or above the 95th percentile of the sex-specific growth charts, and overweight if they are between the 85th and 95th percentiles.^50^ BMI is defined as an individual’s body weight divided by the square of his or her height. Although it is not a measure of actual body fat, it can be calculated by using callipers to measure three skin folds, then plugging those measurements into sex-specific equations. BMI is widely accepted as an accurate proxy for body fat percentage in the general adult population, and it is the measurement of choice in the scientific literature on obesity. © Coneyl Jay/Photo Researchers, Inc.

The concept of obesogens has spread into the public awareness, too, with documentaries such as “Programmed to be Fat?” which aired on the Canadian Broadcasting Corporation (CBC) Network in January 2012 and a session on obesogens at the Society of Environmental Journalists annual conference in October 2011.^14^

## Multiple Modes of Action

The main role of fat cells is to store energy and release it when needed. Scientists also now know that fat tissue acts as an endocrine organ, releasing hormones related to appetite and metabolism. Research to date suggests different obesogenic compounds may have different mechanisms of action, some affecting the number of fat cells, others the size of fat cells, and still others the hormones that affect appetite, satiety, food preferences, and energy metabolism.^15^ Some obesogenic effects may pass on to later generations through epigenetic changes, heritable modifications to DNA and histone proteins that affect when and how genes are expressed in cells, without altering the actual genetic code.^15^^,^^16^^,^^17^

Bruce Blumberg, a biology professor at the University of California, Irvine, coined the term “obesogen” in 2006 when he discovered that tin-based compounds known as organotins predisposed laboratory mice to gain weight.^18^ “If you give tributyltin [TBT] to pregnant mice, their offspring are heavier than those not exposed,” he says. “We’ve altered the physiology of these offspring, so even if they eat normal food, they get slightly fatter.”

Human exposure and health-effect data are relatively rare for organotins, but studies have documented the presence of these compounds in human blood,^19^ milk,^20^ and liver^21^ samples. Although phased out as a biocide and marine antifouling agent, TBT is still used as a wood preservative and, along with dibutyltin, as a stabilizer in polyvinyl chloride; it pollutes many waterways and contaminates seafood.^22^

Blumberg was studying endocrine disruptors in the early 2000s when he heard at a meeting in Japan that TBT causes sex reversal in multiple fish species. “I decided to test whether TBT activated known nuclear receptors, expecting it to activate a sex steroid receptor,” Blumberg says. Instead, it activated peroxisome proliferator–activated receptor gamma (PPARγ), the master regulator of adipogenesis, the process of creating adipocytes, or fat cells.^23^ PPARγ is evolutionarily conserved between mice and humans, and it may be particularly susceptible to chemical “imposters” because it has a large ligand-binding pocket that can accommodate many chemical structures. When a molecule capable of activating the receptor enters the pocket, it turns on the adipogenic program.

“If you activate PPARγ in a preadipocyte, it becomes a fat cell. If it already is a fat cell, it puts more fat in the cell,” Blumberg says. “TBT is changing the metabolism of exposed animals, predisposing them to make more and bigger fat cells.” PPARγ selectively causes multipotent stromal cells to differentiate into bone or fat, and Blumberg found TBT exposure caused these stem cells to show an increased commitment to becoming adipocytes at the expense of the bone lineage. “The insidious thing is that our animals are exposed *in utero* to TBT, then never again, yet TBT caused a permanent effect.”

## A Growing List of Potential Obesogens

Obesity is strongly linked with exposure to risk factors during fetal and infant development.^15^ “There are between fifteen and twenty chemicals that have been shown to cause weight gain, mostly from developmental exposure,” says Jerry Heindel, who leads the extramural research program in obesity at the National Institute of Environmental Health Sciences (NIEHS). However, some obesogens have been hypothesized to affect adults, with epidemiologic studies linking levels of chemicals in human blood with obesity^24^ and studies showing that certain pharmaceuticals activate PPARγ receptors.^15^^,^^25^

Chemical pesticides in food and water, particularly atrazine and DDE (dichlorodiphenyldichloroethylene—a DDT breakdown product), have been linked to increased BMI in children and insulin resistance in rodents.^26^^,^^27^ Certain pharmaceuticals, such as the diabetes drug Avandia® (rosiglitazone), have been linked to weight gain in humans and animals,^9^^,^^17^ as have a handful of dietary obesogens, including the soy phytoestrogen genistein^28^ and monosodium glutamate.^15^

Most known or suspected obesogens are endocrine disruptors. Many are widespread,^29^ and exposures are suspected or confirmed to be quite common. In one 2010 study, Kurunthachalam Kannan, a professor of environmental sciences at the University at Albany, State University of New York, documented organotins in a designer handbag, wallpaper, vinyl blinds, tile, and vacuum cleaner dust collected from 24 houses.^30^ Phthalates, plasticizers that also have been related to obesity in humans,^31^ occur in many PVC items as well as in scented items such as air fresheners, laundry products, and personal care products.

One of the earliest links between human fetal development and obesity arose from studies of exposure to cigarette smoke *in utero*.^32^^,^^33^ Although secondhand-smoke exposure has decreased by more than half over the past 20 years, an estimated 40% of nonsmoking Americans still have nicotine by-products in their blood, suggesting exposure remains widespread.^34^ Babies born to smoking mothers are frequently underweight, but these same infants tend to make up for it by putting on more weight during infancy and childhood.^35^ “If a baby is born relatively small for its gestational age, it tries to ‘play catch-up’ as it develops and grows,” explains Retha Newbold, a developmental biologist now retired from the NTP.

This pattern of catch-up growth is often observed with developmental exposure to chemicals now thought to be obesogens, including diethylstilbestrol (DES), which Newbold spent the last 30 years studying, using mice as an experimental model. Doctors prescribed DES, a synthetic estrogen, to millions of pregnant women from the late 1930s through the 1970s to prevent miscarriage. The drug caused adverse effects in these women’s children, who often experienced reproductive tract abnormalities; “DES daughters” also had a higher risk of reproductive problems, vaginal cancer in adolescence, and breast cancer in adulthood.^36^ Newbold discovered that low doses of DES administered to mice pre- or neonatally also were associated with weight gain,^37^ altered expression of obesity-related genes,^38^^,^^39^ and modified hormone levels.^38^^,^^39^

“What we’re seeing is there’s not a difference in the number of fat cells, but the cell itself is larger after exposure to DES,” Newbold says. “There was also a difference in how [fat cells] were distributed—where they went, how they lined up, and their orientation with each other. The mechanism for fat distribution and making fat cells are set up during fetal and neonatal life.”

## High-Profile Exposures

Animal studies have also implicated another suspected obesogen: bisphenol A (BPA), which is found in medical devices, in the lining of some canned foods, and in cash register receipts.^40^ “BPA reduces the number of fat cells but programs them to incorporate more fat, so there are fewer but very large fat cells,” explains University of Missouri biology professor Frederick vom Saal, who has studied BPA for the past 15 years. “In animals, BPA exposure is producing in animals the kind of outcomes that we see in humans born light at birth: an increase in abdominal fat and glucose intolerance.”

Many endocrine disruptors exhibit an inverted U-shaped dose–response curve, where the most toxic response occurs at intermediate doses.^41^ However, in a recent unpublished study, vom Saal found that BPA affected rodent fat cells at very low doses, 1,000 times below the dose that regulatory agencies presume causes no effect in humans, whereas at higher doses he saw no effect. Receptors typically respond to very low levels of hormone, so it makes sense that they may be activated by low levels of an endocrine mimic, whereas high levels of a chemical may actually cause receptors to shut down altogether, preventing any further response.^41^ This is known as “receptor downregulation.” As a result, some endocrine disruptors have greater effects at low than at high doses; different mechanisms may be operating.^15^

Still another widespread obesogen is perfluorooctanoic acid (PFOA), a potential endocrine disruptor and known PPARγ agonist.^42^ “Pretty much everyone in the U.S. has it in their blood, kids having higher levels than adults, probably because of their habits. They crawl on carpets, on furniture, and put things in their mouth more often,” explains NIEHS biologist Suzanne Fenton. PFOA is a surfactant used for reduction of friction, and it is also used in nonstick cookware, Gore-Tex™ waterproof clothing, Scotchgard™ stain repellent on carpeting, mattresses, and microwavable food items. In 2005 DuPont settled a class-action lawsuit for $107.6 million after its factory outside Parkersburg, West Virginia, tainted nearby drinking water supplies with PFOA.^43^ In December 2011 an independent science panel found the first “probable link” between PFOA and a human health outcome, pregnancy-induced hypertension^44^ (for more information, see “Pregnancy-Induced Hypertension ‘Probably Linked’ to PFOA Contamination,” p. A59 this issue^45^).

Fenton studied how PFOA levels similar to those in the tainted drinking water affected the hormone levels and weight of rodent offspring exposed *in utero*.^46^ “We gave pregnant mice PFOA only during pregnancy. It has a long half-life, so it hangs around during lactation and gets delivered in milk to babies,” Fenton says. “Once the offspring reached adulthood, they became obese, reaching significantly higher weight levels than controls.”

Exposed offspring also had elevated levels of leptin, a hormone secreted by adipose tissue that affects appetite and metabolism. Leptin normally suppresses appetite, but obese people and animals have elevated leptin levels, leading researchers to suspect the brain can become resistant to its effects.^47^ Fenton did not observe weight gain when mice were exposed to PFOA as adults, although her team did find abnormalities in the uterus and mammary gland in exposed adults.

## Eye on Prevention

If exposure during pregnancy predisposes people to gain weight, can diet and exercise ultimately make any difference? Blumberg does not consider the situation hopeless. “I would not want to say that obesogen exposure takes away free will or dooms you to be fat,” he says. “However, it will change your metabolic set points for gaining weight. If you have more fat cells and propensity to make more fat cells, and if you eat the typical high-carbohydrate, high-fat diet we eat [in the United States], you probably will get fat.”

Blumberg postulates that the effects of early-life exposure are irreversible, and those people will fight a life-long battle of the bulge. However, if such people reduce their exposure to obesogens, they will also reduce health effects that may arise from ongoing adulthood exposures. Blumberg believes it’s good to reduce exposure to all kinds of endocrine-disrupting chemicals. “Eat organic, filter water, minimize plastic in your life,” he says. “If there’s no benefit and some degree of risk, why expose yourself and your family?”

Heindel hopes the NIH’s new grant-making effort will yield important discoveries. “It’s a very new field, and people are always skeptical of new fields,” he says. “It’s up to us to get more data to show that chemicals are actually interfering with the endocrine system that controls weight gain and metabolism. And there’s still the question of how important is this to humans. We’re never going to know until we get more data.”

**Figure:**
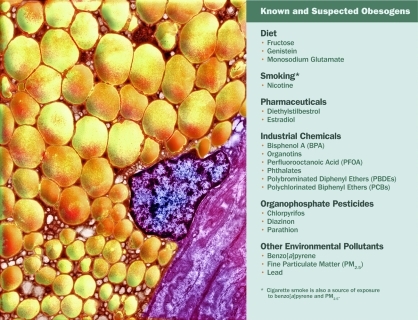
Transmission electron micrograph of human fat cells. Research to date suggests that different obesogens may have different mechanisms of action, affecting either the number or size of fat cells or the hormones that affect appetite, satiety, food preferences, and metabolism. © David M. Phillips/Photo Researchers, Inc.

**Figure:**
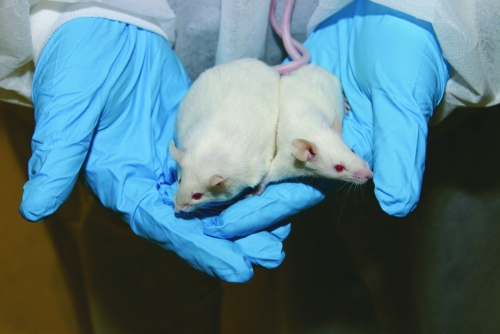
In one study by NIEHS biologist Suzanne Fenton, mice exposed prenatally to PFOA were more likely than controls to become obese when they reached adulthood.^46^ Christopher G. Reuther/EHP

“What if this was really true and chemicals are having a significant effect on obesity?” muses Heindel. “If we could show environmental chemicals play a major role, then we could work on reducing exposure during sensitive windows, and that could have a huge effect [on obesity prevalence].” It would change the focus from treating adults who are already obese to preventing obesity before it starts—a fundamental shift in thinking about obesity.

The NIEHS is crafting priorities for research on potential obesogens. Thayer was the primary force behind the workshop “The Role of Environmental Chemicals in the Development of Diabetes and Obesity,”^48^ held in January 2011 and cosponsored by the NTP, the Environmental Protection Agency, and the Food and Drug Administration National Center for Toxicology Research. “The idea was to have the experts look through the literature and see which might be the most compelling signals, and which areas were emerging but warranted more research,” Thayer explains. These findings will help identify priorities for future research, and a ser ies of papers from the workshop are being submitted for publication.

“We were surprised at the number of chemicals that seem to be interacting with signaling pathways involved in weight regulation,” Thayer says. She adds that evidence also suggests these same compounds are linked with diabetes and metabolic syndrome, “an understudied but natural research direction that brings together the obesity and diabetes issues.”
